# Kinetically Equivalent Functionality and Reactivity of Commonly Used Biocompatible Polyurethane Crosslinking Agents

**DOI:** 10.3390/ijms22084059

**Published:** 2021-04-14

**Authors:** Lajos Nagy, Bence Vadkerti, Csilla Lakatos, Péter Pál Fehér, Miklós Zsuga, Sándor Kéki

**Affiliations:** 1 Department of Applied Chemistry, Faculty of Sciences and Technology, University of Debrecen, Egyetem tér 1, H-4032 Debrecen, Hungary; nagy.lajos@science.unideb.hu (L.N.); vadkerti.bence@science.unideb.hu (B.V.); lakatoscsilla@science.unideb.hu (C.L.); zsuga.miklos@science.unideb.hu (M.Z.); 2 Research Centre for Natural Sciences, Magyar Tudósok Körútja 2., H-1117 Budapest, Hungary; feher.peter@ttk.mta.hu

**Keywords:** glycerol, sorbitol, sucrose, phenyl-isocyanate, reactivity, kinetics

## Abstract

In this paper, the kinetics of the reaction of phenyl isocyanate with crosslinking agents such as sucrose, sorbitol, and glycerol are reported. Crosslinking agents were used in high molar excess to isocyanate to obtain pseudo-first-order rate dependencies, and the reaction products were separated by high-performance liquid chromatography and detected by UV spectroscopy and mass spectrometry. It was found that the glycerol’s primary hydroxyl groups were approximately four times reactive than the secondary ones. However, in the case of sorbitol, the two primary OH groups were found to be the most reactive, and the reactivity of hydroxyl groups decreased in the order of k_OH(6)_(8.43) > k_OH(1)_(6.91) > k_OH(5)_(1.19) > k_OH(2)_(0.98) > k_OH(3)_(0.93) > k_OH(4)_(0.64), where the numbers in the subscript and in the brackets denote the position of OH groups and the pseudo-first-order rate constants, respectively. The Atomic Polar Tenzor (APT) charges of OH groups and dipole moments of monosubstituted sorbitol derivatives calculated by density functional theory (DFT) also confirmed the experimental results. On the other hand, the reactions of phenyl isocyanate with crosslinking agents were also performed using high excess isocyanate in order to determine the number of OH-groups participating effectively in the crosslinking process. However, due to the huge number of derivatives likely formed in these latter reactions, a simplified reaction scheme was introduced to describe the resulting product versus reaction time distributions detected by matrix-assisted laser desorption/ionization mass spectrometry (MALDI-TOF MS). Based on the results, the kinetically equivalent functionality (f_k_) of each crosslinking agent was determined and found to be 2.26, 2.6, and 2.96 for glycerol, sorbitol, and sucrose, respectively.

## 1. Introduction

In recent years, the development and production of polyurethane-based materials (PURs) have been steadily increasing. Due to their versatility and easy-to-tailor properties, the application of PURs spans from electronic components to various biomedical devices [1,2]. Furthermore, they have been frequently used polymeric materials in tissue engineering as well [3,4]. It is, however, essential in biomedical applications that the material planned to be embedded into the body be biocompatible. Thus, the most straightforward synthetic strategies to create various biocompatible PURs are the ones that utilize biocompatible building components [5,6].

Furthermore, in order to obtain PURs with improved mechanical properties, crosslinks must be formed between the polymer chains [7,8]. Several types of compounds, for example, amines (e.g., triethanolamine) [9], thiols (e.g., trimethylolpropane tris(3-mercaptopropionate)) [10] or alcohols (e.g., glycerol [11] are frequently used as crosslinking agents. Thus, alcohols with more than two OH groups can be appropriate crosslinkers for polyurethanes. As the crosslink density is greatly dependent on the number of reacting groups of the crosslinker, biomolecules with more reactive OH groups such as glycerol, sorbitol, and sucrose [12] are an excellent choice for crosslinking reactions yielding highly crosslinked, potentially biocompatible PUR products. However, the reactivities of the OH groups towards isocyanate, due to their different chemical environments, are evidently different, which, in turn, will affect the resulting crosslinking efficiency and density [13,14,15]. Consequently, the knowledge of the reactivity of each OH group in these biomolecules is essential to fully understand and describe the crosslinking process. For example, sucrose, which has eight hydroxyl groups, can be used as a crosslinking agent. However, it has been shown that the increasing amount of sucrose can decrease crosslink density since the reaction between isocyanate and sucrose occurs mainly on primary hydroxyl groups in positions 6 and 6′, resulting in long polymer chains with lower crosslink density [16].

To the best of our knowledge, no detailed report on the kinetic studies of the reaction of glycerol, sorbitol, and sucrose with isocyanate has been reported except for the latter, which was studied only with excess sucrose [16]. Thus, our aim was to determine the reactivity of each hydroxyl group for glycerol, sorbitol, and sucrose towards phenyl isocyanate used as a model compound, and hence to be able to find the most reactive functional groups of these materials that are responsible for forming crosslinks in polyurethane networks.

## 2. Results and Discussion

### 2.1. Reactions with High Excess Molar Crosslinking Agent

To determine the pseudo first-order rate constants, the reaction products were separated by HPLC-UV, and the areas under the detected peaks in the UV chromatogram were used for the calculation of the mole fractions of reaction products (Equation (1)). In order to obtain pseudo-first-order rate dependencies, all crosslinking agents were used in high molar excess relative to phenyl isocyanate. In line with our previous results [17,18], it was found that the molar absorption coefficient of aromatic carbamates (urethane) was independent of the reacting alcohols; hence, the UV peak area ratios reflect the molar ratios of the corresponding reaction products directly.

(1) $$X_{i}=\frac{A_{i}}{\sum_{i=0}^{n}A_{i}}=A_{r,i}$$

where X_i_, A_i_, and A_r,i_ are the mole fraction, the UV peak area of the i^th^ compound, and the relative UV peak area of the i^th^ compound, respectively.

**Figure 1:**
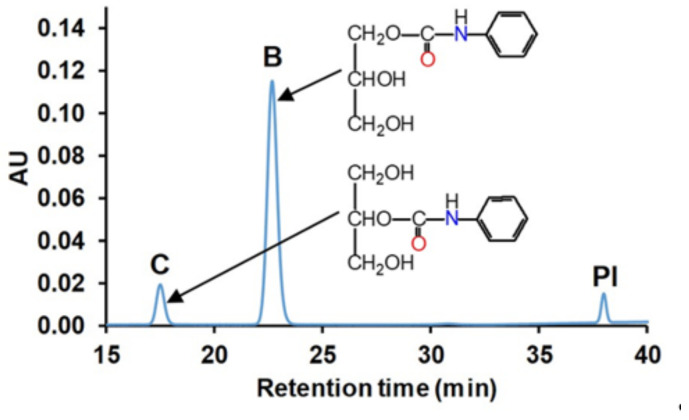
HPLC-UV chromatogram of the methanol-quenched reaction mixture of phenyl isocyanate (PI) with glycerol recorded at λ = 233 nm using C-18 column (the gradient method is shown in Table S1). The reaction time was 14 min. The detected peaks B and C correspond to the structures shown in the Figure inset.

For instance, the mole fraction of product B in Figure 1, which shows a representative chromatogram for the separation of reaction products obtained from the reaction between glycerol and phenyl isocyanate, can be calculated by Equation (2)

(2) $$A_{r,B}=\frac{A_{B}}{A_{C}+A_{B}+A_{\mathrm{PI}}}$$

where A_r,B_ is the relative UV peak area of B compound, A_B_, A_C_ and A_PI_ are the UV peak area of B, C and PI, respectively.

As seen in Figure 1, the two main reaction products were formed. The peak with higher intensity (B) could be attributed to the reaction product formed by the reaction between the primary hydroxyl group of glycerol with phenyl isocyanate, while peak C corresponded to the product obtained from the reaction of secondary OH and PI. The variations of the relative peak areas of the reaction products can be described by Equations (3)–(5)

(3) $$A_{r,B}\left(t\right)=\frac{{2k}_{1}}{{2k}_{1}+k_{2}}\left[1-e^{-\left({2k}_{1}+k_{2}\right)t}\right]$$

(4) $$A_{r,C}\left(t\right)=\frac{k_{2}}{{2k}_{1}+k_{2}}\left[1-e^{-\left({2k}_{1}+k_{2}\right)t}\right]$$

(5) $$A_{r,\mathrm{PI}}\left(t\right)=1-A_{r,B}\left(t\right)-A_{r,C}\left(t\right)$$

where k_1_ and k_2_ are the pseudo first-order rate constants of the formation of B and C products, respectively.

**Figure 2:**
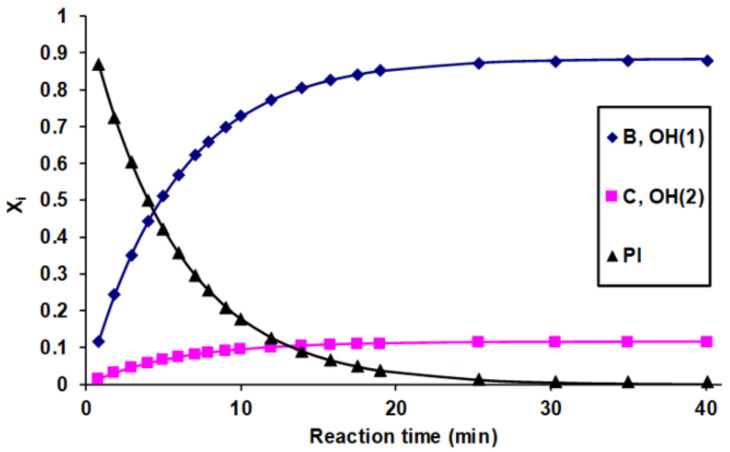
Variations of the molar ratios with time for the phenyl isocyanate and glycerol derivatives substituted at the OH(1) and OH(2) positions. The symbols and the solid lines represent the experimental data and the fitted curves, respectively. Experimental conditions: (glycerol)_0_ = 0.16 M, (phenyl isocyanate)_0_ = 0.01 M in DMSO, and temperature = 30 °C.

Equations (3)–(5) were applied to determine the corresponding pseudo-first-order rate constants by fitting them to the experimental data, as shown in Figure 2.

As seen in Figure 2, the kinetics of the reaction of glycerol with phenyl isocyanate could unambiguously be described by Equations (3)–(5). The determined values of the pseudo-first-order rate constants are shown in Table 1.

As it turns out from Table 1, the rate constant of the reaction of the primary hydroxyl group was 3.8 times higher than that of the secondary hydroxyl group, in good agreement with our previous results obtained for the reaction of diphenylmethane-diisocyanate (MDI) isomers with primary and secondary alcohols [18].

In addition to glycerol, different polyols and carbohydrates are also frequently used as crosslinking agents in polyurethane chemistry. Comparing the rate constants obtained previously for the most reactive primary OH groups of sucrose (0.0445 min^−1^ and 0.0372 min^−1^ for the 6′ and 6 OH, respectively) [13] with primary OHs of the glycerol (0.0762 min^−1^, Table 1), it could be surmised that the reactivity of the glycerol primary OHs was much higher than that of the sucrose under the same experimental conditions (e.g., temperature, the solvent used, etc.). This finding is most likely due to the dissimilar molecular environment of the reacting OH groups, such as the presence of glucose and fructose rings in sucrose. In addition, the differences in the reactivities were more pronounced for the secondary OHs, i.e., secondary OH groups of glycerol reacted approximately 6–10 times faster than those of sucrose.

**Figure 3:**
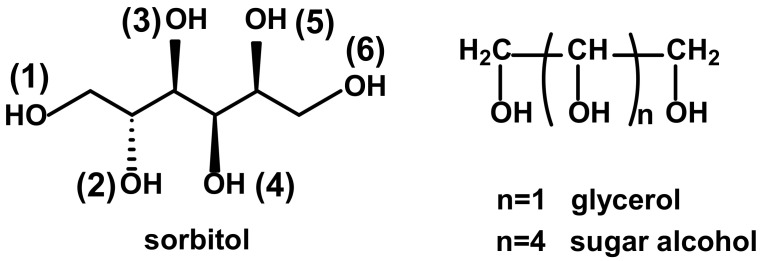
Structures of sorbitol and polyols.

Sorbitol (can be formally derived from the glycerol as seen in Figure 3) is a typical sugar alcohol, which is also a frequently used crosslinking agent and/or initiator in the PU-polyol chemistry. Thus, to get a deeper insight into the reactivities of its primary and secondary OH groups, kinetic studies utilizing phenyl isocyanate reactions were performed.

**Figure 4:**
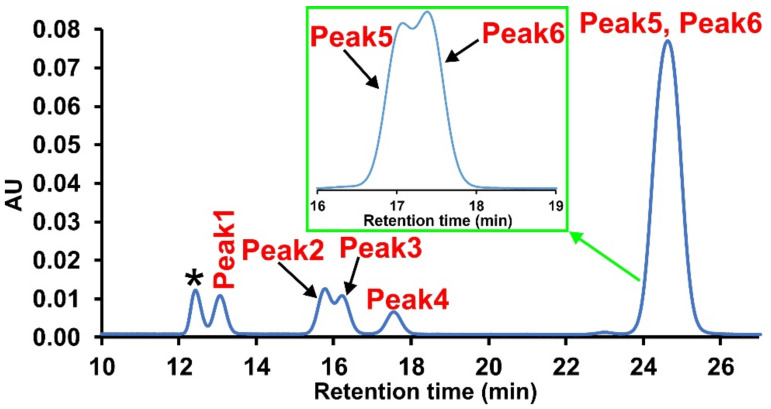
HPLC-UV chromatogram for the sorbitol-PI reaction products recorded at λ = 233 nm, the reaction time was 14 min. The separation of the compounds was achieved using a C18-PFP column; the inset shows a better separation for Peak 5 and Peak 6 products using a different HPLC gradient method (Tables S3 and S4). The asterisk denotes the aniline formed from phenyl isocyanate in the presence of water, which cannot be eliminated completely despite the applied fresh, anhydrous DMSO, and the dry nitrogen or argon atmospheres.

In Figure 4 a representative HPLC-UV chromatogram is shown for the products obtained from the reaction of sorbitol with phenyl isocyanate.

As shown in Figure 4, six different peaks were attributed to the products of the reaction of OH groups of the sorbitol with PI were observed. Although baseline separations of Peak 2, Peak 3, and Peak 5, Peak 6 reaction products could not be achieved. Deconvolution of the peaks allowed the determination of the individual peak areas to calculate the corresponding mole fractions as shown above (see Equation (1)). The variations of the mole fractions of the products can be expressed by Equations (6) and (7)

(6) $$A_{r,i}\left(t\right)=\frac{k_{i}}{\sum_{i=1}^{n}k_{i}}\left(1-e^{-\sum_{i=1}^{n}k_{i}t}\right)$$

(7) $$A_{r,\mathrm{PI}}\left(t\right)=1-\sum_{i=1}^{n}A_{r,i}\left(t\right)$$

where A_r,i_ is the mole fraction (area fraction) of the i^th^ reaction product (Peak 1–Peak 6 products as shown in Figure 4) and k_i_ (from k_1_ to k_6_ according to the order of appearance of peaks) is the rate constant of an individual OH group in the sorbitol.

**Figure 5:**
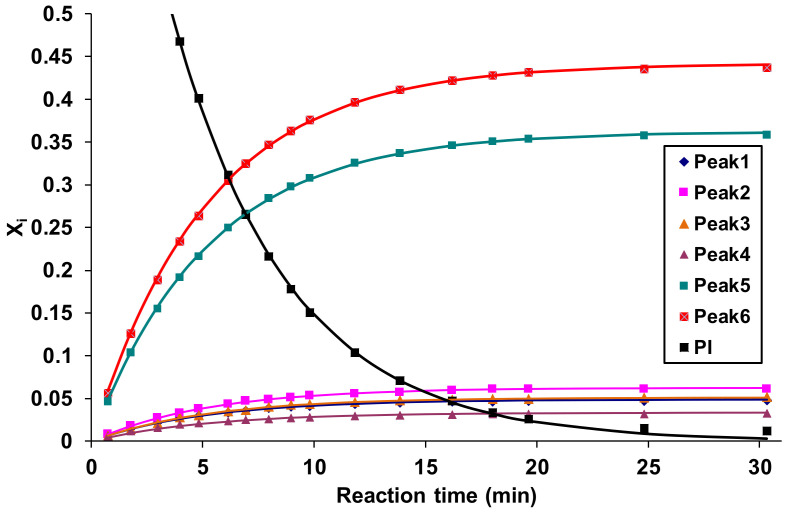
Variations of the mole ratios with time for the phenyl isocyanate and sorbitol derivatives related to Peak 1–Peak 6 shown in Figure 4. The symbols and the solid lines represent the experimental data and the fitted curves, respectively. Experimental conditions: (sorbitol)_0_ = 0.16 M, (phenyl isocyanate)_0_ = 0.01 M in DMSO, and temperature = 30 °C.

The corresponding pseudo-first-order rate constants were determined by means of Equations (6) and (7) by fitting them to the experimental data, as shown in Figure 5.

The rate constants for sorbitol obtained from the fittings are summarized in Table 2.

Based on the data of Table 2, it can be concluded that: (i) k_5_ and k_6_ were significantly higher than the other rate constants for sorbitol, indicating that k_5_ and k_6_ could unambiguously be assigned to the primary hydroxyl groups; (ii) no significant difference was found in the reactivity of primary OH groups of sorbitol and glycerol and, one of the primary OH in sorbitol had a slightly higher and the other one a slightly lower reactivity than glycerol’s primary OH group. Interestingly, the average value of k_5_ and k_6_ was approximately equal to the value of k_1_ for glycerol); (iii) based on the pseudo-first-order rate constant ratios, it could also be surmised that the difference in the reactivity of the primary and secondary hydroxyl groups was higher for the sorbitol then for the glycerol indicating that the reactivity of the secondary OH groups decreased as *n* increased (see Figure 3 for *n*). Furthermore, it is interesting to note that the ratio of the pseudo-first-order rate constants for the primary OH groups in sorbitol (i.e., k_5_/k_6_ = 0.82) was close to that was observed previously for the 6 and 6′ OH groups of sucrose (i.e., k_OH (6)_/k_OH (6′)_ = 0.84). On the other hand, the pseudo-first-order rate constants for the primary OH groups in both sorbitol and glycerol were found to be significantly higher than those for sucrose [13].

In order to attempt to assign the rest of the pseudo-first-order rate constants to the corresponding OH group of sorbitol, density functional theory (DFT) calculations were performed to calculate the Atomic Polar Tenzor (APT) charges of the oxygen atoms in the sorbitol molecule. The APT charges were determined for two conformers available in the literature [19]. Taking into consideration that nucleophilic addition takes place between the isocyanate and the OH group, it is reasonable to assume that the higher the extent of the negative charge on the OH group, the higher the reactivity towards phenyl isocyanate.

**Figure 6:**
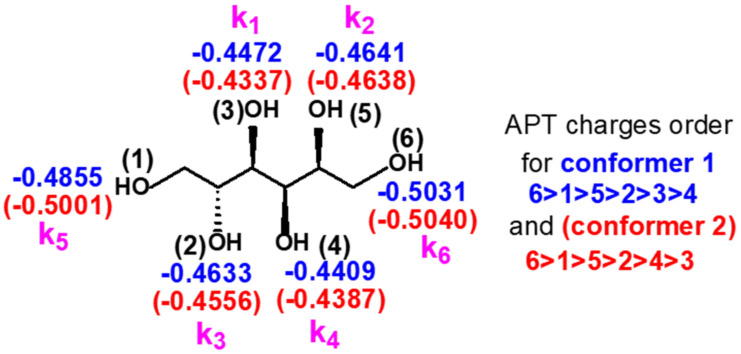
Assignments of the rate constants (numbered by order of appearance of related peaks in Figure 4 (pink)) for two conformers (conformer 1 (blue), conformer 2 (red)) together with the atomic polar tenzor (APT) charges calculated by density functional theory (DFT).

**Figure 7:**
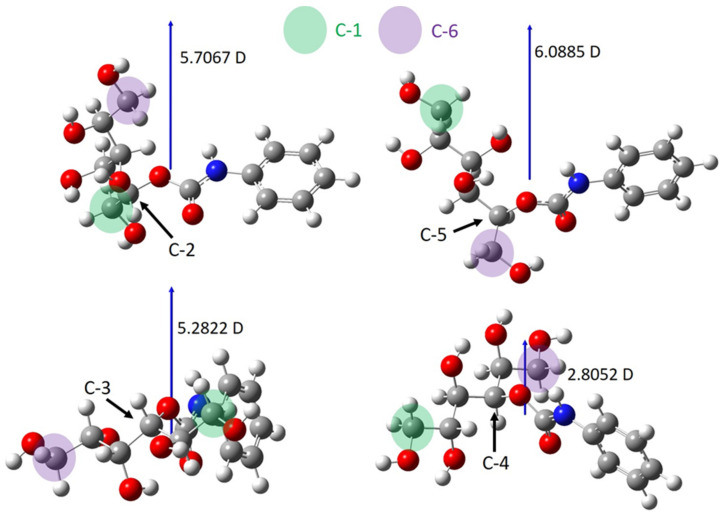
The dipole moment of the isomers formed by the reaction of secondary hydroxyl groups and phenyl isocyanate.

Figure 6 reveals that according to the calculations, the most negative partial charges are on the oxygen atoms of OH(1) and OH(6) in both conformers suggesting that these primary OH groups may have the highest reactivity among all the OH groups in sorbitol. This finding is in line with the data reported in the PUR literature, i.e., the reactivity of the primary OH group is higher than that of the secondary one [17,20,21]. Based on the calculated APT charges, k_5_ and k_6_ can be assigned to OH(1) and OH(6), respectively. Furthermore, as shown in Figure 6, the order of the APT charges of the first four OH groups was the same for both conformers, thus based on this finding, k_2_ and k_3_ could be rendered to OH(5) and OH(2), respectively (the third and the fourth-highest rate constants). On the other hand, it could also be expected, due to the very similar polarity, that the reaction products of the OH group pairs OH(1)-OH(6), OH(2)-OH(5), OH(3)-OH(4), i.e., those located at the same distance from the ends of sorbitol, can hardly be separated by HPLC. In addition, it was found previously for the reaction products obtained from the reaction between the hexanol isomers and isocyanate that using the same (or very similar) chromatographic conditions for their separations; the retention decreased in the order of 1-hexanol > 2-hexanol > 3-hexanol [18]. Based on these results, the peaks in Figure 4 could be assigned as follows: Peak 5 and Peak 6 can unambiguously be attributed to the primary OH groups (at positions one and six, see Figure 6), Peak 2 and Peak 3 were assigned to the OH groups at positions five and two, while the third double peak at ca. 13 min retention time should be rendered to OH(3) and OH(4). Nevertheless, Peak 1 and Peak 4 could not be simply assigned using only the APT charges because, as shown in Figure 6, the partial charges for the OH(3) and OH(4) were very close to each other and were different for the two conformers. Moreover, as seen in Figure 4, Peak 1 and Peak 4 could easily be separated, and this finding could be interpreted by taking into consideration the thinking presented above. Hence, in order to interpret the relatively high difference in the retention times of the products formed by the reactions of OH(3) and OH(4) with phenyl isocyanate, the dipole moments of the urethane isomers formed with the secondary OH groups were calculated (Figure 7).

In Figure 7, the numbering of the carbon atoms corresponds to that of the OH groups presented in Figure 6 (e.g., OH(1) bonded to C(1)). Figure 7 revealed no significant differences in the dipole moments for C(2), C(3), and C(5) isomers; however, the dipole moment of the C(4) isomer where the phenyl isocyanate reacted with the OH(4) hydroxyl group was significantly lower (2.8052 D) than that of the other three isomers (5.2–6.1 D). This could affect the retention time observed in the HPLC chromatogram using a reverse phase C_18_ column. Of course, it is evident that the retention time (under the same chromatographic conditions) could also be influenced by several other parameters such as the position of the OH group reacted with the phenyl isocyanate, conformation of the isomer, dipole moment, and so on. Nevertheless, it can be surmised from the significantly lower dipole moment for the C(4) isomer that this isomer may have the highest retention time among those shown in Figure 7. Thus, Peak 4 and Peak 1, i.e., k_4_ and k_1_ could be assigned to OH(4) and OH(3), respectively, and the reactivity of the hydroxyl groups in sorbitol decreased in the following order: OH(6) > OH(1) > OH(5) > OH(2) > OH(3) > OH(4).

### 2.2. Reactions with High Excess Molar Isocyanate

**Figure 8:**
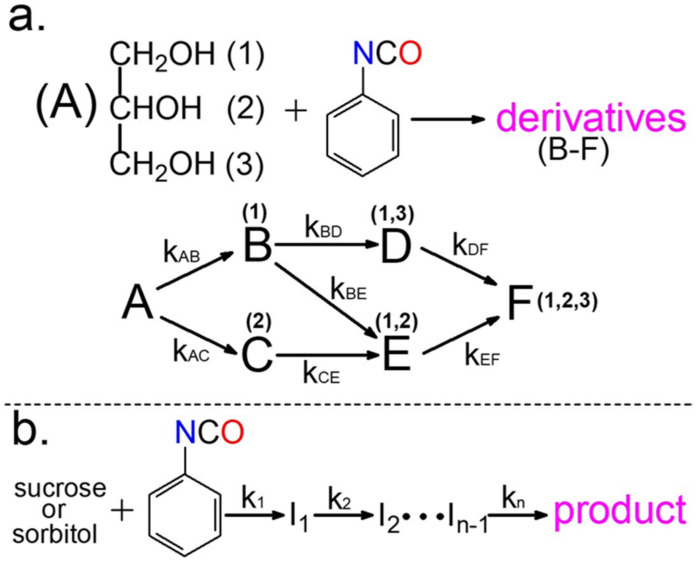
Kinetic scheme for the reaction of glycerol (**a**), sucrose, and sorbitol (**b**) with phenyl isocyanate in the presence of excess molar isocyanate.

The results obtained from the reactions performed in the presence of excess crosslinking agent are useful to determine the relative reactivities of the OH groups on the one hand, but on the other hand, it is also important to know how many OH groups can indeed take part in the crosslinking process. The reactivity of the free (unreacted) OH groups can significantly vary if some OH groups in the crosslinking agent have been reacted with isocyanate. To simulate the process that happens to the crosslinking agent during the formation of crosslinks, the reactions were performed by applying high excess molar phenyl isocyanate. As seen in Figure 8a, in the case of the glycerol (that was the simplest crosslinking agent among the alcohols studied), the kinetics of the reaction becomes more complicated with respect to those observed in the presence of excess alcohol.

**Figure 9:**
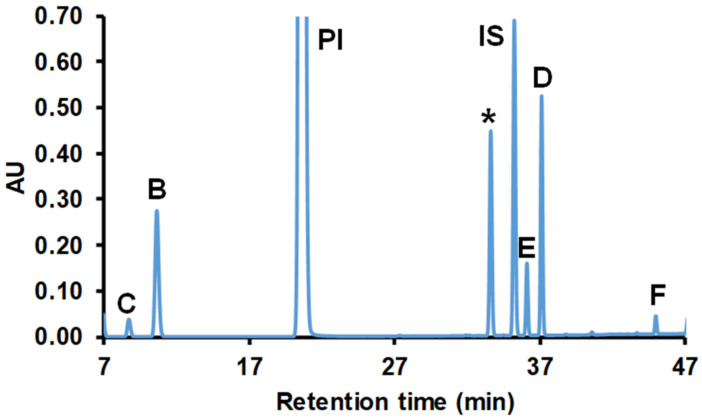
HPLC-UV chromatogram of the products formed by the reaction of glycerol and phenyl isocyanate (PI) recorded at λ = 233 nm and reaction time of 14 min (the parameters for the applied gradient method are compiled in Table S2). IS = internal standard phenyl isocyanate blocked by propanol, and the asterisk denote the N,N diphenyl carbamide formed from the aniline and phenyl isocyanate (it was unambiguously identified by HPLC-MS).

Figure 8a shows the reaction of the first OH group with isocyanate that yields derivatives B and C (with the same structures as shown in Figure 1). The numbers in the brackets above the letters denote the OH groups of glycerol reacting with phenyl isocyanate, while k represents the corresponding pseudo-first-order rate constants. The reaction of derivative B with phenyl isocyanate gave two additional, disubstituted derivatives D and E. However, it was evident that E was also formed from C and that derivatives D and E yielded the trisubstituted derivative F (final product). As demonstrated in Figure 9, each derivative formed during the reaction was successfully baseline separated.

**Figure 10:**
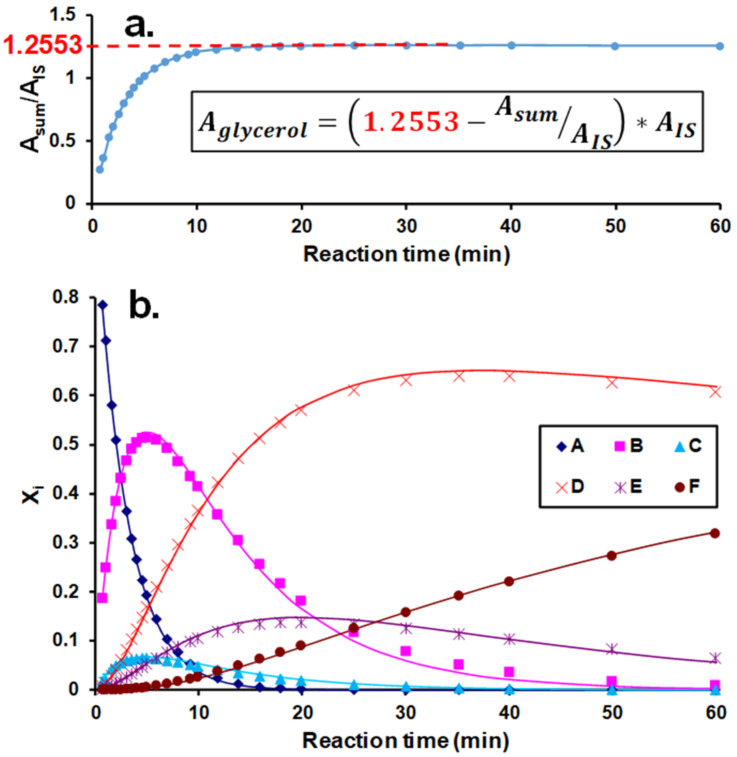
Variation of the ratio of the total peak area (A_sum_) to the internal standard peak’s area (A_IS_) with the reaction time and the calculation of the area for the unreacted glycerol (**a**) and the product distributions as a function of time for the reaction of glycerol with phenyl isocyanate (**b**). Experimental conditions: (glycerol)_0_ = 0.0067 M, (phenyl isocyanate)_0_ = 0.45 M in DMSO, and temperature = 30 °C.

The mole fractions of the derivatives were determined using the peak areas obtained from the UV chromatogram similar to those discussed previously. Moreover, under the present experimental conditions, derivatives with a number of PI moieties spanning from 1 to 3 (such as D, E, and F) could be identified. Thus, the peak areas obtained from the UV chromatograms of these derivatives were divided by the number of PI moieties present in the corresponding derivative (supposing that the molar extinction coefficient was proportional to the number of PI moieties). Furthermore, glycerol (A) could not be detected as it has no absorption in the UV range. Moreover, in order to determine the mole fraction of the unreacted glycerol, internal standard (IS, phenyl isocyanate blocked by propanol) was added to the reaction mixture. As demonstrated in Figure 10a, by taking into account the total UV peak area of the derivatives (A_sum_) and plotting the ratio of A_sum_ to A_IS_ (UV peak area of the internal standard) as a function of reaction time, the A_sum_/A_IS_ ratio remained constant after ca. 15 min reaction time. This finding clearly indicates that the glycerol was consumed within the 15 min reaction time, and the total peak area of the derivatives remained constant afterward. Furthermore, this latter finding also demonstrates that absorption of the derivatives was indeed proportional to the number of PI moieties.

Using the constant value for the ratios A_sum_/A_IS_, the A_glycerol_ were calculated by the formula shown in the inset of Figure 10a and the corresponding area fractions (A_r,X_) (mole fractions) were determined by Equation (8).

(8) $$A_{r,X}=\frac{A_{X}/n}{A_{\mathrm{glycerol}}+A_{\mathrm{sum}}}$$

where A_X_ is the peak area for derivatives B, C, D, E and F (i.e., X = B, C, D, E, and F), while *n* is the number of phenyl isocyanate attached to glycerol, i.e., *n* = 1 for derivatives B and C, *n* = 2 for derivatives D and E, and *n* = 3 in the case of derivative F and A_sum_ = A_B_ + A_C_ + A_D_ / 2 + A_E_ / 2 + A_F_ / 3.

The variations of the relative peak areas of the glycerol and reaction products can be described by Equations (9)–(14).

(9) $$A_{r,A}\left(t\right)=e^{-\left({2k}_{\mathrm{AB}}+k_{\mathrm{AC}}\right)\cdot t}$$

(10) $$A_{r,B}\left(t\right)=\frac{{2k}_{\mathrm{AB}}}{{2k}_{\mathrm{AB}}+k_{\mathrm{AC}}-k_{\mathrm{BD}}-k_{\mathrm{BE}}}\left[e^{-\left(k_{\mathrm{BD}}+k_{\mathrm{BE}}\right)\cdot t}-e^{-{(2k}_{\mathrm{AB}}+k_{\mathrm{AC}})\cdot t}\right]$$

(11) $$A_{r,C}\left(t\right)=\frac{k_{\mathrm{AC}}}{{2k}_{\mathrm{AB}}+k_{\mathrm{AC}}-{2k}_{\mathrm{CE}}}\left[e^{-{2k}_{\mathrm{CE}}\cdot t}-e^{-{(2k}_{\mathrm{AB}}+k_{\mathrm{AC}})\cdot t}\right]$$

(12) $$\begin{array}{l} A_{r,D}\left(t\right)=\frac{{2k}_{\mathrm{AB}}\cdot k_{\mathrm{BD}}}{{(2k}_{\mathrm{AB}}+k_{\mathrm{AC}}-k_{\mathrm{BD}}-k_{\mathrm{BE}}{)(k}_{\mathrm{BD}}+k_{\mathrm{BE}}-k_{\mathrm{DF}})}\left[e^{-k_{\mathrm{DF}}\cdot t}-e^{-{(k}_{\mathrm{BD}}+k_{\mathrm{BE}})\cdot t}\right]+\\ \frac{{2k}_{\mathrm{AB}}\cdot k_{\mathrm{BD}}}{{(2k}_{\mathrm{AB}}+k_{\mathrm{AC}}-k_{\mathrm{BD}}-k_{\mathrm{BE}}{)(2k}_{\mathrm{AB}}+k_{\mathrm{AC}}-k_{\mathrm{DF}})}\left[e^{-{(2k}_{\mathrm{AB}}+k_{\mathrm{AC}})\cdot t}-e^{-k_{\mathrm{DF}}\cdot t}\right] \end{array}$$

(13) $$\begin{array}{l} A_{r,E}\left(t\right)=\left(\frac{a_{2}}{a_{3}-a_{1}}-\frac{a_{2}}{a-4a_{1}}+\frac{a_{5}}{a_{6}-a_{1}}-\frac{a_{5}}{a_{4}-a_{1}}\right)\cdot e^{-a_{1}t}-\frac{a_{2}}{a_{3}-a_{1}}\cdot e^{-a_{3}t}+\frac{a_{2}}{a_{4}-a_{1}}\cdot e^{-a_{4}t}-\\ \frac{a_{5}}{a_{6}-a_{1}}\cdot e^{-a_{6}t}+\frac{a_{5}}{a_{4}-a_{1}}\cdot e^{-a_{4}t} \end{array}$$

(14) $$A_{r,F}\left(t\right)=1-A_{r,A}\left(t\right)-A_{r,B}\left(t\right)-A_{r,C}\left(t\right)-A_{r,D}\left(t\right)-A_{r,E}\left(t\right)$$

where

$$\begin{array}{l} a_{1}=k_{\mathrm{EF}}{;\;a}_{2}=\frac{{2k}_{\mathrm{AB}}k_{\mathrm{BE}}}{{2k}_{\mathrm{AB}}+k_{\mathrm{AC}}-k_{\mathrm{BD}}-k_{\mathrm{BE}}}{;\;a}_{3}=k_{\mathrm{BD}}+k_{\mathrm{BE}}{;\;a}_{4}={2k}_{\mathrm{AB}}+k_{\mathrm{AC}};\\ a_{5}=\frac{k_{\mathrm{AC}}{2k}_{\mathrm{CE}}}{{2k}_{\mathrm{AB}}+k_{\mathrm{AC}}-{2k}_{\mathrm{CE}}}{;\;a}_{6}={2k}_{\mathrm{CE}} \end{array}$$

where k_AB_, k_AC_, k_BD_, k_BE_, k_CE,_ k_EF_ are pseudo first order rate constants shown in Figure 8a.

As shown in Figure 10b, Equations (9)–(14) adequately describe the experimental kinetic data. Hence, the pseudo-first-order rate constants shown in Figure 8a could be determined, and these values are compiled in Table 3.

Based on the data of Table 3., it can be concluded that the value of the ratio of k_AB_ and k_AC_ (3.94) is very similar to that obtained from the pseudo-first-order rate constants of primary and secondary OHs (3.83) in the case of excess molar glycerol. These data indicate that the relative reactivity of OH groups in the glycerol does not change significantly whether glycerol or phenyl isocyanate was applied in excess. Furthermore, from the data in Table 3, it can be seen that the value of k_AB_ was significantly higher than those of k_CE_ and k_BD_, showing that the reactivity of the primary OH group of glycerol decreases (negative substitution effect) upon the reaction of the other with PI (the same trend was valid for the secondary OH group, i.e., k_AC_ > k_BE_). The decrease in the reactivities could be interpreted by the negative inductive, and steric effects in the derivatives C and B, and these effects are more pronounced if the PI moiety was in the vicinity of the unreacted OH group (i.e., k_CE_ < k_BD_). Finally, it also was evident from Table 3 that the reactivity of the OH groups was remarkably reduced with the increasing number of the PI moiety. For example, the order of k_AC_ > k_BE_ > k_DF_ (for secondary OH) and k_AB_ > k_BD_ > k_EF_ or k_AB_ > k_CE_ > k_EF_ (for primary OH) could be established.

As it was pointed out earlier, due to the formation of various intermediates, the kinetics of the reaction became very complicated in the presence of excess isocyanate. This was even more evident for crosslinking agents with many OH groups. For instance, in the case of sorbitol, 64 different derivatives (including the unreacted sorbitol, i.e., $\sum_{m=0}^{n}\left(\begin{array}{c} n\\ m \end{array}\right)$ is equal to 2^n^, where n is the total number of OH groups, while m is the number of OH groups that have been reacted) could be formed during the reaction. Among these derivatives, lots of isomers could also be formed. For example, in the case of sorbitol containing three phenyl isocyanate moieties, the number of isomers is $\left(\begin{array}{c} 6\\ 3 \end{array}\right)$; hence, altogether, 20 isomers are possible in this case. For sucrose, the situation is even more complicated with the potential presence of 256 possible derivatives in the reaction mixture. Thus, it is clear that, due to the huge number of derivatives most likely formed during the reaction, the reaction products could not be separated by HPLC, and hence, the reaction system could not be treated similarly to that presented for glycerol.

**Figure 11:**
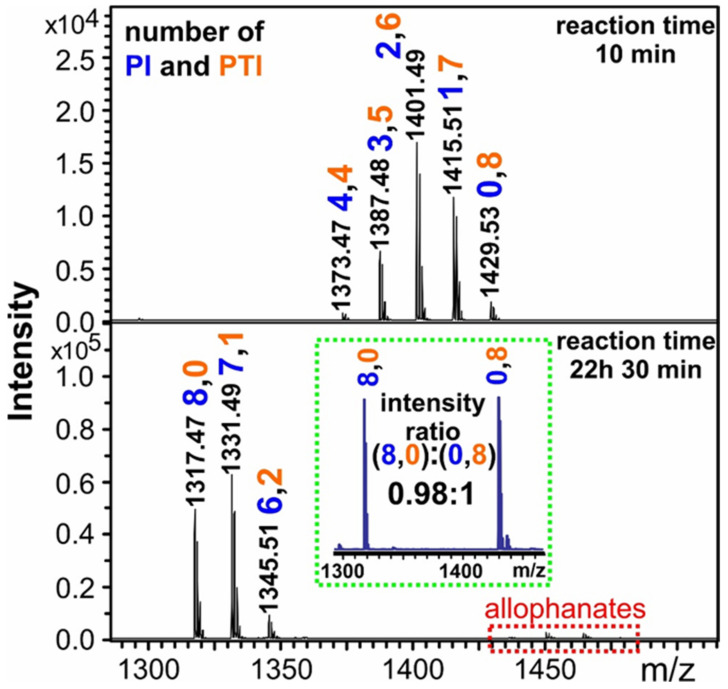
MALDI-TOF MS spectra of the reaction mixture of sucrose and phenyl isocyanate (PI) recorded at 10 min and 1350 min reaction times after derivatization with p-tolyl isocyanate (PTI). The numbers above the mass peaks represent the number of PTI and PI units attached to the sucrose. The inset shows the spectrum of sucrose derivatized completely with PI (8,0) and PTI (0,8) at 1:1 molar ratio. During PTI derivatization, negligible allophanate formation was observed.

To handle this problem, it was supposed that the isomers formed during the reaction should be considered together without separation in each reaction step, and the kinetics of the reaction were then simplified to a consecutive “gross” reaction as shown in Figure 8b. Furthermore, MALDI-TOF MS was chosen for the identification and quantification of intermediates (I_1_, I_2_, etc., in Figure 8b). However, the possible differences in the ionization efficiencies of the various intermediates did not allow the direct conversion of the MS intensity ratios to the corresponding molar ratios (e.g., the ionization efficiency of sucrose was significantly lower than those of the derivatives formed with the sequential addition of PI moieties). In addition, due to the significant mass differences between the reaction products formed (e.g., M = 342 Da for sucrose and M = 1294 Da for sucrose with eight PI), mass discriminations occur; hence, the intensity ratios will not reflect the corresponding molar ratios. Thus, to overcome the difficulties associated with the varying ionization efficiencies and mass discrimination effect, samples collected at predetermined reaction times were quenched by methanol, followed by removing the solvents in a vacuum. The unreacted OH groups in sucrose were then derivatized with p-tolyl isocyanate (PTI). The MALDI-TOF MS spectra of the reaction mixtures of sucrose and phenyl isocyanate after derivatization with PTI are shown in Figure 11.

Sucrose was derivatized separately with phenyl isocyanate and with p-tolyl isocyanate. After derivatization, the two sucrose derivatives were mixed in 1:1 molar ratio. As seen in the inset of Figure 11, the MALDI-MS intensity ratio was approximately 1:1, indicating that there was no significant variation in the ionization efficiencies for the two derivatives (in the Figure 11 inset they are labeled as 8,0 and 0,8) on the one hand, and the mass discrimination was negligible, on the other hand. Furthermore, the standard deviation of the MALDI-MS intensity ratio was lower than 10%. If the intensity ratios detected in the MALDI spectrum indeed reflect the molar ratios for these derivatives, where formally all the eight PI moieties were substituted by PTIs, it is reasonable to assume that the intensity ratios will reflect the molar ratios as well for all the derivatives formed after PTI derivatization. Therefore, the experimental mole fractions were calculated from the corresponding MALDI-MS intensity fractions similarly to those shown previously for the HPLC measurements using the peak area fractions. For example, the experimental mole fraction of the product with *m*/*z* 1401 is shown in the upper panel of Figure 11 (at 10 min reaction time) was calculated by Equation (15).

(15) $$X_{2,6}=\frac{I_{2,6}}{I_{4,4}+I_{3,5}+I_{2,6}+I_{1,7}+I_{0,8}}=I_{r,2}$$

The variations of the relative MALDI-MS intensities of the reaction products can be described by Equations (16)–(18). Equation (17) is known as the Bateman equation and is commonly used for the description of consecutive reactions [22]

(16) $$I_{r,0}\left(t\right)=I_{r,\mathrm{sucrose}}\left(t\right)=e^{-k_{1}t}$$

(17) $$I_{r,n}\left(t\right)=\sum_{i=1}^{n+1}\left[\left(\prod_{j=i}^{n}k_{j}\right)\cdot \left(\sum_{j=i}^{n+1}\left(\frac{e^{-k_{j}t}}{\prod_{p=i,p\neq j}^{n+1}{(k}_{p}-k_{j})}\right)\right)\right]$$

(18) $$I_{r,8}\left(t\right)=1-I_{r,\mathrm{sucrose}}\left(t\right)-\sum_{l=1}^{n}I_{r,n}\left(t\right)$$

where n is the number of PI moieties, and it spans from *n* = 1 to 7.

**Figure 12:**
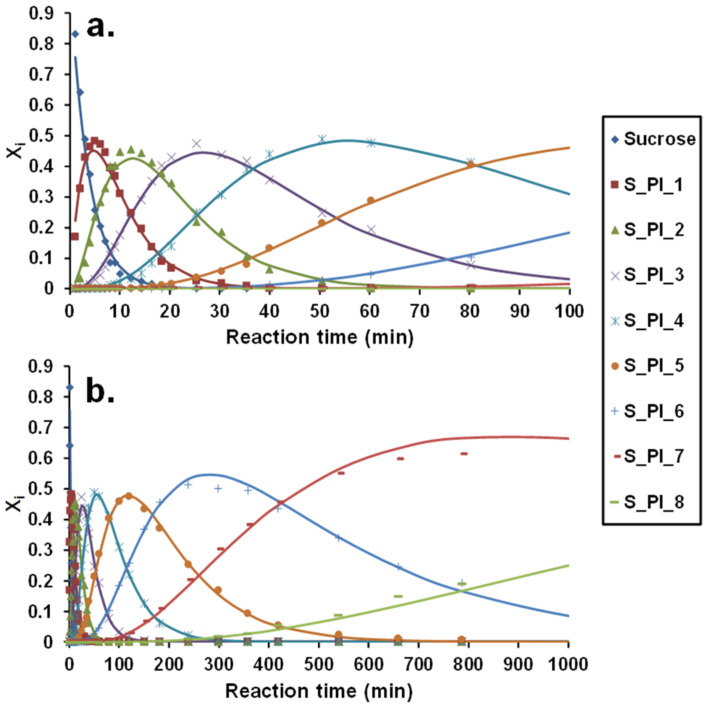
The product distributions as a function of time (**a**) 0–100 min; (**b**) 0–1000 min for the reaction of sucrose with phenyl isocyanate. Experimental conditions: (sucrose)_0_ = 0.0025 M, (phenyl isocyanate)_0_ = 0.45 M in DMSO, and temperature = 30 °C.

From Figure 12, it can be seen that Equations (16)–(18) adequately describe the experimental kinetic data. The determined pseudo-first-order rate constants are summarized in Table 4.

The pseudo-first-order rate constants for sorbitol were determined similarly, and the corresponding pseudo-first-order rate constants are compiled in Table 5. The experimental kinetic data together with the fitted curves can be found in the Supplementary Information Figure S1.

It can be surmised from the data in Table 4 that the values of the pseudo-first-order rate constants significantly decreased as the number of PI moieties attached to the crosslinking agent increased. Furthermore, assuming equivalent reactivity for all the eight OH groups in sucrose, the average reactivity of an OH group for the formation of monosubstituted derivative could be calculated as k_1_/8 = 0.0328 min^−1^. Hence, the pseudo-first-order rate constant with the lowest value (k_8_) was only 1.8% of the value of k_1_/8. It was found that the pseudo-first-order rate constant of the most reactive OH group of sucrose was higher by approximately one order of magnitude than that of the least reactive OH group applying excess sucrose [13]. However, in the presence of high excess isocyanate, this difference is ca. two orders of magnitude. This latter finding may indicate that the negative inductive and/or steric effects (negative substitution effect) are enhanced by increasing the PI moieties in sucrose. A similar trend was obtained for the values of the pseudo-first-order rate constants for sorbitol.

### 2.3. Evaluation of the Pseudo First-Order Rate Constants and Determination of Kinetically Equivalent Functionality

It is worth discussing the pseudo-first-order rate constants for the reactions carried out with excess crosslinking agent (CA) and phenyl isocyanate (PI). For the reactions with excess CA, a concentration of 0.16 M CA was applied, while in the presence of excess PI, the PI concentration was 0.45 M. Supposing that the reaction rate depends on the first orders with respect to both PI and CA (i.e., second-order kinetics is assumed), the pseudo-first-order rate constants for the formation of monosubstituted derivative (containing one PI moiety) can now be compared by taking into consideration the concentration of the reactant applied in excess. For example, the gross pseudo-first-order rate constant (observed) for excess glycerol (0.16 M) can be calculated using the sum of the corresponding pseudo-first-order rate constants as k = 2k_1_ + k_2_ = 0.1721 min^−1^, while that of glycerol in the presence of excess PI ((PI)_0_ = 0.45 M) can also be given as 2k_AB_ + k_AC_ = 0.3306 min^−1^. To compare the two rate constant values, the concentrations of the reactants applied in excess should be taken into consideration, as shown by Equation (19)

(19) $$k_{\mathrm{corr}}=k\frac{{\left[\mathrm{CA}\right]}_{0}}{{\left[\mathrm{PI}\right]}_{0}}$$

where k_corr_ is the concentration corrected pseudo-first-order rate constant, k is the gross pseudo-first-order rate constants obtained with excess CA, [CA]_0_ and [PI]_0_ are the initial concentrations of CA and PI, respectively.

According to Equation (19), the k_corr_ for glycerol can be given as 0.3306/0.45*0.16 = 0.1175 min^−1^. The rate constants obtained for sorbitol and sucrose were calculated similarly, and the corresponding values of the rate constants are summarized in Table 5.

As can be seen in Table 5, the rate constants of glycerol and sorbitol were higher than that of sucrose, indicating that the OH groups attached to the sucrose rings possess lower reactivity. On the other hand, the rate constants for the reaction with PI excess are lower than those determined in the presence of CA excess, and the ratio of k_corr_/k is approximately 0.7 in all cases. The difference in the values of rate constants may be originated from the fact that the first-order dependence of the reaction rate supposed for both reactants was not entirely valid. Furthermore, in the case of excess PI, the polarity of the reaction medium is likely different from that of the reaction mixture containing excess CA.

The kinetically equivalent functionality (f_k_) from the pseudo-first-order rate constants for the reactions performed with phenyl isocyanate excess can be calculated by Equation (20):

(20) $$f_{k}=\frac{\sum_{i=1}^{n}\frac{k_{i}}{n-i+1}}{\frac{k_{1}}{n}}$$

where n is the number of the OH groups in the crosslinking agent, k_i_ is the total pseudo-first-order rate constant for the crosslinking agent in each reaction step (i.e., k_1_ is the total rate constant of the formation of the monosubstituted derivative from the crosslinking agent, k_2_ is the gross rate constant of the formation of disubstituted derivative from the monosubstituted intermediate, etc.).

It is important to note that in the presence of excess isocyanate, the pseudo-first-order rate constants for the reactions of each individual OH group with phenyl isocyanate could not be determined in the case of sucrose and sorbitol (as discussed before). Nevertheless, as we have shown, they were successfully determined for glycerol (Table 2). Thus, k_1_, k_2_ and k_3_ for glycerol in Equation (20) were calculated as k_1_ = 2k_AB_ + k_AC_, k_2_ = k_BD_ + k_BE_ + 2k_CE_, etc.).

On the other hand, the kinetically equivalent functionality could also be determined from the pseudo-first-order rate constants for the reaction taking place in the presence of excess crosslinking agent. In this reaction, under these conditions, only monosubstituted products are formed, and the value of f_k_ can be calculated by Equation (21)

(21) $$f_{k}=\frac{\sum_{i=1}^{n}k_{i}}{k_{\max}}$$

where ∑k_i_ is the sum of the pseudo-first-order rate constants obtained for the OH groups of the crosslinking agent, k_max_ is the pseudo-first-order rate constants of the most reactive OH group.

Using Equations (20) and (21) the f_k_ values could be calculated for both reaction systems, and they are compiled in Table 6.

From Table 6, it can be seen that there was no significant difference in the kinetically equivalent functionalities whether they were obtained from the data of the reaction systems with excess CA or excess PI. Furthermore, higher kinetically equivalent functionality was obtained for glycerol than for sorbitol in spite of the higher functionality of the latter. Furthermore, sorbitol can be regarded as a bifunctional reactant (chain extender) in the first reaction step and monofunctional in the second reaction step. Using k_1_/2 and k_2_ in Equation (20) (instead of k_1_/6 and k_2_/5), the obtained value for f_k_ was a little bit higher than 2 (2.02). However, this value indicates that due to the very low reactivities of the secondary OH groups, the kinetically equivalent functionality was close to 2 for sugar alcohols. Finally, the kinetically equivalent functionality determined for sucrose was higher than that of the other two crosslinking agents, which can be interpreted by the presence of three primary OH groups in the sucrose molecule and the higher functionality of sucrose.

## 3. Materials and Methods

### 3.1. Chemicals

Phenyl isocyanate (98%) (PI), p-tolyl isocyanate (PTI), dimethyl sulfoxide (DMSO, anhydrous, 99.9%), D-sorbitol (98%), and glycerol (99%), tin(II) 2-ethylhexanoate (TEH) and toluene were purchased from Sigma-Aldrich (Darmstadt, Germany) and all were used as received except the toluene, which was purified and dried according to the well-known procedure [23]. The D(+)-sucrose (puriss, Ph. Eur. 6.) was obtained from Reanal (Budapest, Hungary) and dried in a vacuum oven at 40 °C for overnight before use. Methanol (HPLC grade) from Merck (Darmstadt, Germany) was used without any purification.

### 3.2. Kinetic Studies in the Presence of Crosslinking Agent Excess

Into a vial thermostated at 30 °C, dry DMSO and calculated amount of crosslinking agent were added under nitrogen atmosphere to obtain a solution (V = 10 mL) at a concentration of 0.16 M. Then, 10 μL of phenyl isocyanate (PI) was injected into the solution to a concentration of 0.01 M. For the high-performance liquid chromatography (HPLC) measurements, after predetermined time intervals, 50 μL of samples were taken out from the reaction mixture and added to 950 μL methanol to quench the unreacted phenyl isocyanate. These quenched samples were used for HPLC measurements without further sample preparation.

### 3.3. Kinetic Studies in the Presence of Excess Isocyanate 

In 9 mL dry DMSO (placed in a vial thermostated at 30 °C), phenyl isocyanate (PI) was dissolved followed by the addition of 1 mL DMSO solution of the crosslinking to get 10 mL reaction mixture at concentrations of 0.45 M and 0.02 M for the PI and the total OH group concentration of the crosslinking agent, respectively. After predetermined time intervals, 50 μL of samples were taken out from the reaction mixture, and 100 μL methanol (950 μL in case of the glycerol) was added to quench the free isocyanate. The quenched glycerol samples were studied by HPLC. In the case of sucrose and sorbitol, the quenched samples were stored under vacuum at room temperature for 24 h to remove the methanol content. Then the free, unreacted OH groups were capped with p-tolyl isocyanate (PTI) (60 mg/mL) in the presence of tin(II) 2-ethylhexanoate (TEH) (20 mg/mL) catalyst using 100 μL DMSO/Toluene 1/1 (*v*/*v*) as a solvent; the reaction time was 24 h. The samples were then quenched by 100 μL methanol and analyzed by matrix-assisted laser desorption/ionization (MALDI) mass spectrometry.

### 3.4. High-Performance Liquid Chromatography (HPLC)

A 10 μL sample from the solution prepared by diluting 50 μL of the reaction mixture with methanol to 1000 μL was injected into a chromatographic system consisting of a Waters 2695 Separations Module equipped with a thermostable autosampler (5 °C), a column module (45 °C), a Waters 2996 photodiode array detector (PDA), a VDSphere PUR C18-M-SE column (4.6 × 150 mm, 5 μm) (VDS optilab Chromatographietechnik GmbH, Berlin, Germany) and an ACE Excel 5 C18-PFP column (separation of products obtained from the PI-sorbitol reaction, 4.6 × 150 mm, 5 μm) (Advanced Chromatography Technologies Ltd., Aberdeen, UK). The gradients used for the analysis of the products formed in the reactions are summarized in Tables S1–S4. The reaction products were detected with a photodiode array detector in the range from 200 nm to 350 nm, and a flow rate of 1.0 mL/min was applied in all cases.

### 3.5. Matrix-Assisted Laser Desorption/Ionization Time-of-Flight Mass Spectrometry (MALDI-TOF MS)

The MALDI-TOF MS measurements (monitoring the reactions of PI and sorbitol/sucrose in the presence of PI excess) were carried out with a Bruker Autoflex Speed mass spectrometer (Bruker Daltonik, Bremen, Germany) operating in the reflectron mode. For all the measurements 19 kV (ion source voltage 1), 16.65 kV (ion source voltage 2), 21 kV (reflector voltage 1) and 9.55 kV (reflector voltage 2) voltages were used. The solid phase laser (355 nm) was operated at 500 Hz with 60–70% laser attenuation, and 2000 shots were summed. The spectra were externally calibrated using polyethylene glycol standards (M_n_ = 1000 g/mol and 1500 g/mol). The samples were prepared with 2,5-dihydroxybenzoic acid (DHB, 20 mg/mL) and sodium trifluoroacetate (NaTFA, 5 mg/mL) dissolved in a mixture of methanol and distilled water (80/20 *v*/*v*). The solutions were mixed in a 100:1:10 (*v*/*v*/*v*) ratio (matrix/analyte/cationization agent). From these solutions, aliquots of 0.5 μL were deposited onto a metal sample plate and allowed to air-dry.

### 3.6. Density Functional Theory (DFT) Calculations

The APT and dipole moments for sorbitol and its isocyano derivative in DMSO were calculated using DFT via the M06 functional [24] and the def2-TZVP basis set [25,26]. The initial geometry, obtained from the Biological Magnetic Resonance Data Bank [19], was reoptimized inside the solvent cavity, which was modeled implicitly with the SMD (Solvation model based on Density) method [27]. The calculations were performed with the Gaussian 16 software package [28].

## 4. Conclusions

The reactions between the commonly used crosslinking agents and phenyl isocyanate were studied in the presence of high molar excess of the reactant to obtain pseudo-first-order kinetics. Applying glycerol and sorbitol in high molar excess to phenyl isocyanate (the reactions were monitored by HPLC-UV), it was found that primary hydroxyl groups were more reactive than the secondary ones (i.e., for glycerol k_1_/k_2_ was determined to 3.83) and no significant difference was observed in the reactivities of the primary OH groups of glycerol and sorbitol. The pseudo-first-order rate constants of the individual OH groups in sorbitol were assessed. Accordingly, each OH group was assigned to the appropriate rate constants by means of the corresponding APT charges on the oxygen atom of the OH groups and the dipole moment of the monosubstituted derivatives determined by DFT. It was found that groups OH(1) and OH(6) were the most reactive, while the OH(4) was the least reactive group in sorbitol. The reaction of glycerol carried out in the presence of excess phenyl isocyanate was monitored by HPLC-UV. However, separation of the reaction products due to the complex reaction mixture formed was not feasible in the case of sorbitol and sucrose derivatives. Thus, when phenyl isocyanate was applied in high molar excess to sorbitol or sucrose, the reactions were followed by means of MALDI-TOF mass spectrometry; moreover, the isomers were indistinguishable. The reactivity of the OH groups significantly decreased with the increasing number of phenyl isocyanate moiety attached to the crosslinking agent. It was also found that the values of the rate constants were higher with an excess of crosslinking agent, indicating that no single second-order kinetics may be valid and/or due to the different polarities of the reaction media for the two reaction systems. Furthermore, the kinetically equivalent functionality (f_k_), which shows the reactivity of the crosslinking agent with respect to that of the most reactive OH group, was introduced and given for glycerol, sorbitol, and sucrose. Furthermore, no significant differences in the f_k_ values calculated from the pseudo-first-order rate constants of the reactions performed in excess of the crosslinking agent or phenyl isocyanate were found (except for sorbitol). The f_k_ value of glycerol (f_k_ = 2.26) was found to be higher than that of sorbitol (f_k_ = 2.02), while for sucrose, f_k_ was determined to be 2.96.

## Figures and Tables

**Table 1:** The determined pseudo-first-order rate constants for glycerol. Experimental conditions: (glycerol)_0_ = 0.16 M, (phenyl isocyanate)_0_ = 0.01 M in DMSO, and temperature = 30 °C. The standard deviation of the rate constants was lower than 5% in all cases.

Rate Constant	k × 10^2^ (min^−1^)	k_1_/k_2_
k_1_	7.61	3.83
k_2_	1.99	

**Table 2:** The determined pseudo-first-order rate constants for sorbitol. Experimental conditions: (sorbitol)_0_ = 0.16 M, (phenyl isocyanate)_0_ = 0.01 M in DMSO, and temperature = 30 °C. The standard deviation of rate constants was lower than 5% in all cases.

Rate Constant	k × 10^2^ (min^−1^)	k_i_/k_6_
k_1_	0.93	0.11
k_2_	1.19	0.14
k_3_	0.98	0.12
k_4_	0.64	0.08
k_5_	6.91	0.82
k_6_	8.43	1.00

**Table 3:** The determined pseudo-first-order rate constants for the reaction of glycerol with phenyl isocyanate. Experimental conditions: (glycerol)_0_ = 0.0067 M, (phenyl isocyanate)_0_ = 0.45 M in DMSO, and temperature = 30 °C. The standard deviation of the pseudo-first-order rate constants was lower than 10% in all cases.

Rate Constant	k × 10^2^ (min^−1^)
k_AB_	14.67
k_AC_	3.72
k_CE_	5.06
k_BD_	8.50
k_BE_	1.70
k_EF_	3.36
k_DF_	0.37

**Table 4:** Determined pseudo-first-order rate constants for the reaction of sucrose and sorbitol with phenyl isocyanate. Experimental conditions: (sucrose)_0_ = 0.0025 M, (sorbitol)_0_ = 0.0033 M, (phenyl isocyanate)_0_ = 0.45 M (in both cases) in DMSO, and temperature = 30 °C. The standard deviation of the pseudo-first-order rate constants was lower than 15% in all cases.

Rate Constant	k × 10^2^ (min^−1^) (Sucrose)	k × 10^2^ (min^−1^) (Sorbitol)
k_1_	26.26	41.56
k_2_	16.89	19.50
k_3_	9.33	4.45
k_4_	4.66	1.00
k_5_	2.04	0.24
k_6_	0.96	0.07
k_7_	0.32	-
k_8_	0.06	-

**Table 5:** Pseudo-first-order rate constants for glycerol, sorbitol, and sucrose obtained from the reactions carried out in the presence of excess crosslinking agent (excess CA, 0.16 M) and phenyl isocyanate (excess PI, 0.45 M), respectively. The k_corr_ was corrected with the concentrations of excess reactants. * This value was determined previously under the same experimental conditions [13].

Crosslinking Agent (CA)	k × 10^2^ (min^−1^) (Excess CA)	k_corr_ × 10^2^ (min^−1^) (Excess PI)	k_corr_/k
glycerol	17.21	11.75	0.68
sorbitol	19.08	14.78	0.77
sucrose	13.17 *	9.34	0.71

**Table 6:** Kinetically equivalent functionalities for glycerol, sorbitol, and sucrose calculated from the pseudo-first-order rate constants of reactions carried out in the presence of excess crosslinking agent (excess CA) and excess phenyl isocyanate (excess PI), respectively. * This value was calculated from the previously published reaction pseudo-first-order rate constants [13]. The value in the brackets was calculated in a different way.

Crosslinking Agent (CA)	f_k_ (Excess CA)	f_k_ (Excess PI)
**glycerol**	2.26	2.26
**sorbitol**	2.26	1.80 (2.02)
**sucrose**	2.96 *	2.81

## Data Availability

Data is contained within the article or Supplementary Materials.

## Supplementary Materials

The following are available online at https://www.mdpi.com/article/10.3390/ijms22084059/s1, Figure S1: The product distributions as a function of time (a. 0–50 min; b. 0–700 min) for the reaction of sucrose and phenyl isocyanate; Table S1: Gradient table of the HPLC conditions used to separate the products of the reaction of glycerol and phenyl isocyanate; Table S2: Gradient table of the HPLC conditions used to separate the products of the reaction of glycerol and phenyl isocyanate; Table S3: Gradient table of the HPLC conditions used to separate the products of the reaction of sorbitol and phenyl isocyanate; Table S4: Gradient table of the HPLC conditions used for better separation of products S5, S6 in the reaction of sorbitol and phenyl isocyanate.
