# Supplementary material for: Kinetically Equivalent Functionality and Reactivity of Commonly Used Biocompatible Polyurethane Crosslinking Agents
Source: Int J Mol Sci. 2021 Apr 14;22(8):4059. doi: 10.3390/ijms22084059 (PMC8071031; doi:10.3390/ijms22084059)
Supplement: Supplementary file 1 [file ijms-22-04059-s001.pdf]

Supplementary Material

Table S1. Gradient table of the HPLC conditions used to separate the products of the reaction of Glycerol and Phenyl-isocyanate (glycerol excess). Column parameters: 150x4,6 mm VDSphere PUR 100 C18-M-SE, 5◎m

Time (min)	Methanol (%)	Water (%)
0.00	16.0	84.0
30.00	16.0	84.0
50.00	80.0	20.0
60.00	80.0	20.0

Table S2. Gradient table of the HPLC conditions used to separate the products of the reaction of Glycerol and Phenyl-isocyanate (isocyanate excess)

Time (min)	Methanol (%)	Water (%)
0.00	25.0	75.0
15.00	25.0	75.0
30.00	50.0	50.0
40.00	65.0	35.0
50.00	80.0	20.0

Table S3. Gradient table of the HPLC conditions used to separate the products of the reaction of Sorbitol and Phenyl-isocyanate (sorbitol excess). Column parameters: ACE Excel 5 C18-PFP, 150x4,6mm, 5◎m)

Time (min)	Methanol (%)	Water (%)
0.00	12.0	88.0
30.00	12.0	88.0
30.10	70.0	30.0
40.00	70.0	30.0

Table S4. Gradient table of the HPLC conditions used for better separation of products S5, S6 in the reaction of Sorbitol and Phenyl-isocyanate (sorbitol excess). Column parameters: ACE Excel 5 C18-PFP, 150x4,6mm, 5⊚m)

Time (min)	Methanol (%)	Water (%)
0.00	16.0	84.0
30.00	16.0	84.0
50.00	80.0	20.0
60.00	80.0	20.0

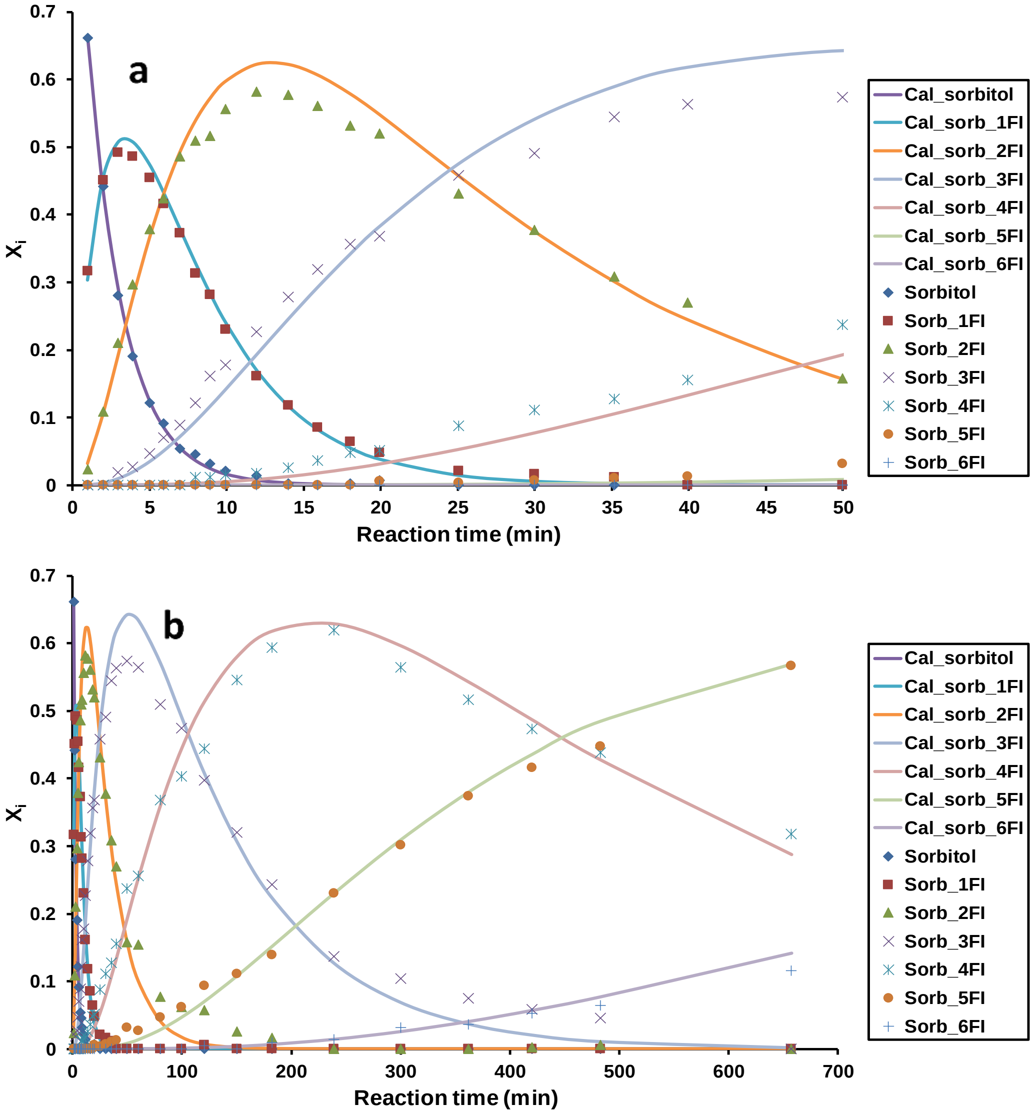

Figure S1. The product distributions as a function of time (a. 0-50 min; b. 0-700 min) for the reaction of sucrose and phenyl isocyanate. Experimental conditions: $[sorbitol]_o$ = 0.0033 M, $[phenyl\ isocyanate]_o$ = 0.45 M in DMSO, temperature = 30 ^oC.
